# Pulmonary function tests and their associated factors in people living with HIV at Jimma medical center; Ethiopia: a comparative cross-sectional study

**DOI:** 10.3389/frph.2023.1178304

**Published:** 2023-10-13

**Authors:** Muluken Teshome Azezew, Teshome Gobena, Misganaw Asmamaw Mengstie, Elias Mulat

**Affiliations:** ^1^ Department of Biomedical Sciences, College of Health Sciences, Debre Tabor University, Debre Tabor, Ethiopia; ^2^ Department of Biomedical Sciences, College of Health Sciences, Jimma University, Jimma, Ethiopia

**Keywords:** HIV, pulmonary function tests, Ethiopia, pulmonary diseases, pulmonary function

## Abstract

**Background:**

People living with HIV (PLHIV) have a greater risk of developing respiratory disorders. The problems are linked to poor socio-economic status, high viral load, low CD4 counts, and antiretroviral therapy. Despite the high prevalence of respiratory disorders, the association between HIV infection and pulmonary function status, as well as the associated factors, is not well established in resource-limited countries.

**Methods:**

A comparative cross-sectional study was conducted from September 24 to October 15 2020 at Jimma Medical Center among people living with HIV who were arranged into an age–sex-matched comparison group. Data were collected using a pretested structured questionnaire administered via face-to-face interviews. The collected data included socio-demographic, respiratory, HIV infection, and substance use variables. Pulmonary function tests were conducted using an SP10 spirometer. The collected data were entered and analyzed using SPSS version 26. Independent t-test and multiple linear regressions were carried out to identify factors associated with the pulmonary function status of the study participants.

**Results:**

A total of 96 PLHIV and 96 matched control individuals participated in the study. The mean of pulmonary function test parameters among the PLHIV respondents was FVC (l) (67.35 ± 19.12, p0.003), FEV1_s_ (l) (61.76 ± 16.04, p0.001), and PEFR (50.14 ± 23.32, p0.001), with a significant lowering in the study group. Female sex, respiratory symptoms, duration of HIV, duration of treatment, and khat chewing were associated with lowered FEV1s (l) (*p* < 0.05) in HIV-positive respondents.

**Conclusion:**

PLHIV had significantly lower mean lung function parameters than HIV-uninfected participants**.** As a result, health providers should screen HIV-positive patients with respiratory symptoms, prolonged duration of HIV infection, prolonged treatment, and khat chewing for non-infectious lung disorders while treating them.

## Introduction

AIDS is a syndrome caused by HIV that predominantly targets and destroys a cluster of differentiation 4 T cells (CD4 cells) (1, 2). It is a pandemic that affects 36 million people worldwide (3).

The lung is a chief target organ for retroviral infection, rendering it vulnerable to an inclusive array of infectious and non-infectious complications (4). Innovation and provision of highly active anti-retroviral treatment (HAART) for PLHIV have been a new horizon in reducing AIDS-related mortality and prolonging life expectancy (5). As a result, the spectrum of infectious and non-infectious diseases is significantly altered (6).

The incidence of non-communicable diseases, particularly chronic non-infectious pulmonary diseases, dramatically increase in PLHIV after HAART (7). Obstructive lung diseases (OLD), especially chronic obstructive pulmonary diseases (COPD) and asthma, are emerging pulmonary burdens (8). Chronic lung diseases are categorized based on the pattern of anomalies detected with pulmonary function test indices.

To date, research is being conducted to confirm the theoretically proposed factors and mechanisms that cause OLD in PLHIV (8). The prevalence of tobacco use in PLHIV is higher than that in the general US population. Despite this, HIV infection is independently associated with OLD incidence and prevalence without smoking exposure. Such a query highlights that OLD in PLHIV is mechanistically beyond the risk factor that has been considered in the general population (5). Altered oral and gut microbiome (9), post-translational modification of *α*_1_-antitrypsin (AAT) (10), and high circulatory HIV-associated markers in plasma such as IL-6, 8, and TNF are some of the postulated mechanisms that initiate altered immune response in the lungs (11).

Socio-demographic factors (age, sex), history of pulmonary tuberculosis, smoking, substance use, time of HAART initiation, CD4 count, viral load, and HAART have been crudely associated with pulmonary disease development and progression in PLHIV (12).

Despite these facts, the screening, diagnosing, and managing of non-infectious chronic lung diseases by interpreting pulmonary function test indices have been under-emphasized in PLHIV (5). Despite the high prevalence and incidence of HIV infection in Ethiopia, there are scarce data to link HIV and pulmonary function test patterns. In this regard, this study will fill a gap by determining pulmonary function tests and associated factors for PLHIV, and the data from this study will be used to establish a routine pulmonary function test platform for PLHIV.

## Methods and materials

### Study area

This study was conducted at Jimma University Medical Centre (JUMC) from September 24 to October 15 2020. JUMC is located in Jimma city, 352 km southwest of Addis Ababa. It provides services for approximately 15,000 inpatients, 160,000 outpatient attendants, 11,000 emergency cases, and 4,500 deliveries a year for patients coming to the hospital from the catchment population of about 15 million people. Currently, 3,029 PLHIV are registered and receive service in the ART clinic.

### Study design

An institution-based comparative cross-sectional study design was used for this study.

### Source population

The source population for the study group was all PLHIV attending the ART clinic at JUMC. The comparison group was HIV uninfected and age- and sex-matched, and it included the caregivers of the attendees >18 years.

### Study population

The study population for the study group comprised all the selected PLHIV attending the ART clinic and the age- and sex-matched HIV-uninfected attendants of age >18 years during the study period. During the matching procedure, we established an age- and sex-matched comparison group for each study group. The age-matching procedure allowed for a two-age deviation.

### Inclusion criteria

The inclusion criteria for the study group were all PLHIV who had begun HAART 6 months before the study period. The comparison group comprised HIV-uninfected and age- and sex-matched individuals, and the caregivers of these PLHIV attendees >18 years were included in the study.

### Exclusion criteria

**Figure 1:**
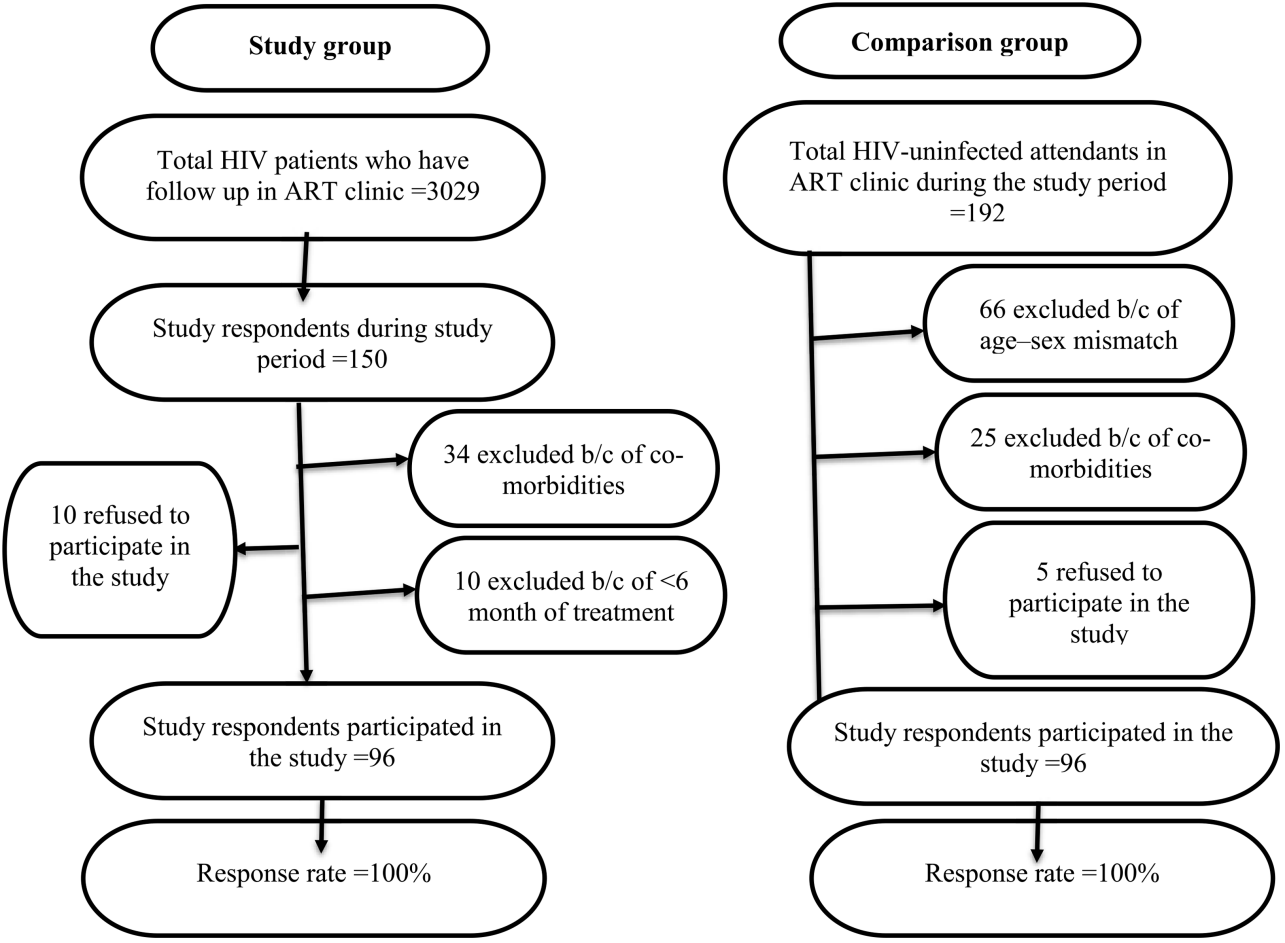
The study respondents were selected based on the inclusion and exclusion criteria of the study, as shown in the flow chart.

PLHIV who had been diagnosed with chronic co-morbidities (hypertension, DM, cancer, PCP, ILD, oral candidiasis, chest deformity, asthma, COPD), pregnancy, <6 months of HAART, severe illness, history of retinal detachment, abdominal surgery <3 months ago, chest/abdominal surgery <3 months ago, and presumed COVID -19 were excluded. Similar exclusion criteria were used for a comparison group. The study respondents were selected based on the inclusion and exclusion criteria of the study, as shown in the flow chart (Figure 1).

### Sample size determination and sampling technique

The sample size was calculated using the double proportion population formula by taking the proportion of obstructive lung disease among PLHIV (P1 = 10%) and HIV-negative respondents (P2 = 3%) extracted from a study conducted in a rural part of South Africa (12). The confidence interval, level of significance, and study power were 95%, 5%, and 90%, respectively. After considering the 10% non-response rate, the total sample sizes were 192 (96 for the study group and 96 for the comparison group). During data collection, both PLHIV and HIV-negative respondents were chosen using a consecutive sampling technique.

### Data collection procedure

Data were collected using an interviewer-administered structured questionnaire. It had five distinct parts: socio-demographic characteristics, a respiratory-related questionnaire, substance use, anthropometry, and spirometer measurements. HIV-related questionnaires were reviewed from the study participants' medical records. Respiratory-related questionnaires were adapted from the St. George's Respiratory Questionnaire (13).

Consented respondents from the comparison group had HIV tests using a rapid HIV diagnostic kit. The procedure and result interpretation were based on the Ethiopian Health and Nutrition Research Institute (14). Weight was measured with the study respondents barefoot and wearing light clothing using a digital weight scale. Height was measured using a stadiometer with the respondents were standing barefoot and with the shoulder in normal alignment. The Medication Adherence Rating Scale (MARS score) was used to assess drug adherence for these PLHIV (15).

### Pulmonary function testing procedure

The pulmonary function tests (PFT) were measured using the SP10 digital spirometer (CONTEC MEDICAL SYSTEMS CO., LTD, China). The PFT parameters measured by the spirometer were forced vital capacity in liter and percent predicted (FVC), forced expiratory volume in the first seconds in liter and percent predicted (FEV1s), FEV1s/FVC, forced expiratory flow at 25%–75% of FVC (FEF_25%–75%_), and peak expiratory flow rate in liter per seconds (PEFR (l/s).

The procedure, precaution, acceptability, reproducibility, and interpretation of the spirometer output were based on the CDC-National Health and Nutrition Examination Survey of Respiratory Health and its research article (16). The criteria for acceptability of PFT were an absence of cough in the first breath and exhalation that should last for six seconds. The criteria for reproducibility were that the two highest values of FVC (l) and FEV_1_ (l) must agree with 150 milliliters (ml).

### Infection control

The body of the spirometer and turbine was cleaned using 70% isopropyl alcohol at the end of each procedure. The data collectors were wearing N-95 facemasks and powder-free disposable gloves after demonstrating the maneuver for the study respondents. Each study participant had a mouthpiece with a bacterial–viral filter, which was discarded at the end of the procedure. In line with COVID-19 prevention, all preventive measures and recommendations from the World Health Organization were kept in place.

### Instrument calibration

The instrument's calibration was based on the SP-10 pocket spirometer user manual that the manufacturer prepared. Under the calibration interface, verification was checked for pre-calibration with a 3l syringe.

Qualified personnel performed a spirometer examination in a quiet room in a sitting position. The procedure was conducted in the morning between 8:00 AM and 12:00 AM daily at room temperature. Each participants' ID, age, sex, weight, height, race, and smoking status were fed into the SP10 spirometer before the maneuver. A very explicit instruction was delivered to the study participants until a common understanding was grounded for the spirometer examination.

### Maneuver

Each study participant was sitting upright with their chin elevated and neck extended slightly, feet flat on the ground with an uncrossed leg, and holding their nostrils tightly by the left/right thumb and index fingers. They then took a big deep breath and filled their lungs with air. Holding the spirometer and placing the mouthpiece into their mouth above the tongue and between the teeth, the participant then blasted out the air as hard and as fast as possible and kept blowing out for the first 6 s to empty the lung. The procedure was repeated until three acceptable and reproducible measurements were obtained, with a maximum of eight attempts. The procedure was stopped and rescheduled if the participant could not produce an acceptable and repeatable spirometer output after eight attempts. Four BSc nurse professionals who had previous experience in data collection collected the data.

### Operational definition

Respiratory symptoms–a Saint George's respiratory questionnaire score >24 is considered a prevalent respiratory symptom.

Alcohol taking–a male drinking ≥ 2 bottles of beer per drinking session and a female drinking ≥ 1 bottle of beer per drinking session over ≥ 3 sessions/week within one month before the study.

Khat chewer–chewing ≥ 1 bundle of khat leaves per chewing session ≥ 3 days/week for one month before the study.

### Data analysis procedure

The collected data were cleaned, coded, entered into epi-data manager (v.4.6.0.2) and epi-data entry client (v.4.6.0.2), and exported into SPSS version 26 for data analysis.

Using SPSS, continuous variables were summarized as mean and standard deviation using descriptive statistics. Categorical variables were summarized as frequency and percentage using cross-tabulation.

An Independent t-test was used to compare the mean of the pulmonary function test indices between the PLHIV and HIV-negative study respondents. The assumption of an independent sample t-test was checked using the Shapiro–Wilk and Levine's tests.

Linear regression was used to investigate the factors that predict FEV1 (l) in PLHIV patients. Variables that were significant predictors of FEV1_s_ (l) in simple linear regression were entered into multiple linear regression (*p* < 0.25).

Multiple linear regression analysis was utilized to obtain the best-fit linear combination of sex, respiratory symptoms, history of pulmonary TB, duration of RVI, duration of treatment, treatment regimen, current CD4 cell count, and khat chewing, which were significant in simple linear regression.

The assumption of multiple linear regression (linearity, normality, homoscedasticity, outliers, and multicollinearity) was checked and met. Normality was checked using histogram, P-P, and Q-Q plots. Multicollinearity was checked using the variance inflation factor (VIF), and VIF >5 shows an association between predictor variables. The recruited variables in multiple linear regression that had a *p*-value of < 0.05 were said to be a significant predictor of FEV1_s_ (l).

### Data quality management

Training for data collectors and supervisors was given by the principal investigator for two consecutive days on the purpose of the study, interview and measurement techniques, and ethical issues. The principal investigator checked for the completeness and clarity of the collected data.

## Results

### Socio-demographic characteristics of the respondents

A total of 192 (96 PLHIV and 96 HIV-negative) were recruited for the study. The response rate of the study respondents was 100%. Among the total respondents, 65% were female. The mean ages of PLHIV and HIV-negative respondents were 37.56 years (SD = ±7.017) and 36.06 years (SD = ±7.20), respectively. Regarding the level of education, 6.3% of PLHIV respondents and 14.6% of HIV-negative respondents were unable to read and write (Table 1), and 9.3% of PLHIV and 30.2% of HIV-negative respondents had a diploma or higher.

**Table 1:** Socio-demographic characteristics of PLHIV and HIV-negative respondents in Jimma town, southwest Ethiopia, 2020.

	PLHIV (*n* = 96)	HIV-negative (*n* = 96)
	mean ± SD	mean ± SD
Demographic characteristics		
Age	37.56 ± 7.02	36.06 ± 7.20
Sex	Number (%)	Number (%)
Female	63 (65.6)	63 (65.6)
Male	33 (34.4)	33 (34.4)
Level of education		Number (%)
Unable to read and write	6 (6.3)	14 (14.6)
Primary	46 (47.9)	31 (32.3)
Secondary	35 (36.5)	22 (22.9)
Diploma or higher	9 (9.3)	29 (30.2)
Occupational status		
Government employer	30 (31.2)	50 (52.1)
Own business	28 (30.4)	32 (34.7)
Daily laborer	25 (26)	11 (11.5)
Unemployed	13 (13.5)	3 (3.1)

SD, standard deviation.

### Anthropometry, medical history, and substance use-related characteristics of respondents

The mean height (meter) of PLHIV and HIV-negative respondents was 1.63 (SD = ±0.86) and 1.65 (SD = ±0.110), respectively. The mean weight (kilogram) of PLHIV and HIV-negative respondents was 59.4 (SD = ±11.81) and 56.91 (SD = ±9.88), respectively. Similarly, the mean BMI (kg/m^2^) of PLHIV and HIV-negative respondents was 21.85 (SD = ±3.57) and 21.49 (SD = ±7.63), respectively. Moreover, 6.8% and 2.6% of PLHIV and HIV-negative respondents reported a history of TB.

In all, 25% of PLHIV respondents were former smokers, and 1% of HIV-negative respondents were current smokers. The proportions of khat chewing and alcohol taking in PLHIV and HIV-negative respondents were 9.4% vs. 11.5% and 11.5% vs. 8.3%, respectively (Table 2).

**Table 2:** Anthropometric, medical history, and substance use-related characteristics of PLHIV and HIV-negative respondents in Jimma town, southwest Ethiopia, 2020.

Variables	PLHIV (*n* = 96)	HIV-negative (*n* = 96)
	mean ± SD	mean ± SD
Height (m)	1.63 ± 0.86	1.65 ± 0.11
Weight (kg)	56.9 ± 9.9	59.42 ± 11.81
BMI (kg/m^2^)	21.85 ± 3.57	21.49 ± 7.63
Medical history	Number (%)	Number (%)
History of PTB	13 (6.8)	5 (2.6)
History of pneumonia	2 (1)	1 (0.5)
Smoking status		
Current smoker	-	2 (2.2)
Former smoker	25 (26)	-
Never smoker	71 (74)	94 (97.8)
Number of cigarettes/day	-	9.5 ± 3.5
Alcohol taking	11 (11.5)	8 (8.3)
Khat chewing	9 (9.4)	11 (11.5)

BMI, body mass index; PTB, pulmonary tuberculosis; SD, standard deviation.

### HIV-related characteristics of the respondents

The mean duration of HIV infection was 7.98 years (SD ± 4.64), and the mean duration of treatment was 7.15 years (SD ± 4.63). The mean of the current CD4 counts and current viral loads was 659 cells/mm^3^ (SD ± 465.53) and 247.32 copies/ml (SD ± 1,036.38), respectively. Regarding the viral load status, 95.8% of PLHIV respondents had a suppressed viral load. Among the virally suppressed PLHIV respondents, 81.5% of them had an undetectable viral load. Using the Medication Adherence Rating Scale (MARS score), it was found that 33.3% of the RVI respondents had poor adherence (Table 3).

**Table 3:** HIV-related characteristics of *PLHIV* in Jimma town, southwest Ethiopia, 2020.

Parameter	PLHIV (mean ± SD)
Duration of HIV (yrs.)	7.98 (4.64)
Duration of treatment (yrs.)	7.15 (4.63)
Current CD4 count (cells/mm^3^)	659 (465.53)
Current viral load (copies/ml)	247.32 (1,036.38)
Drug adherence	Number (%)
High adherence	5 (5.2)
Moderate adherence	59 (61.5)
Low adherence	32 (33.3)

TX, treatment; CD4, cluster of differentiation 4.

### Comparison of mean pulmonary function tests among PLHIV and HIV-negative respondents

The current study showed that the mean of the predicted FVC was significantly lower in PLHIV respondents (67.35 ± 19.12) than in HIV-negative respondents (75.73 ± 18.87, p0.003). Similarly, it revealed that the percentage means of predicted FEV1 (61.76 ± 16.04 vs. 77.1 ± 19.19, p0.001) and PEFR (50.15 ± 23.33 vs. 67.97 ± 25.22, p0.001) were significantly lower in PLHIV than in HIV-negative respondents.

Conversely, the mean score of FEV1/FVC (85.44 ± 45.51 vs. 90.92 ± 42.51, p0.622) and FEF_25–75_ (l/s) (3.14 ± 2.14 vs. 3.33 ± 1.41, p0.733) showed no significant difference between PLHIV respondents and HIV-negative respondents (Table 4).

**Table 4:** Comparison of mean of pulmonary function test indices in *PLHIV* and HIV-negative participants in Jimma town, southwest Ethiopia, 2020.

PFT indices	PLHIV (*n* = 96)	HIV- negative (*n* = 96)	*p*-value
	mean ± SD	mean ± SD	
Height (m)	1.64 ± 0.86	1.65 ± 0.11	0.29
Weight (kg)	56.9 ± 9.9	59 ± 11.8	0.11
BMI (kg/m^2^)	21.85 ± 3.7	21.5 ± 7.6	0.67
FVC(l)	2.96 ± 0.79	3.27 ± 0.77	0.006*
FVC (%)	67.35 ± 19.12	75.73 ± 18.87	0.003*
FEV1(l)	2.29 ± 0.59	2.72 ± 0.74	<0.001*
FEV1 (%)	61.76 ± 16.04	77.10 ± 19.19	0.001*
FEV1/FVC	85.44 ± 45.51	90.92 ± 42.51	0.622
FEF_25−75_ (l/s)	3.14 ± 2.14	3.33 ± 1.41	0.733
PEFR(l/s)	3.33 ± 1.41	4.26 ± 2.04	0.001*
PEFR (%)	50.15 ± 23.33	67.97 ± 25.22	0.001*

Independent sample t-test.

* significant *p*-value, BMI, body mass index; FVC, forced vital capacity; FEV1, forced expiratory volume in the 1st second; FEF_25−75_, mid-forced expiratory flow; PEFR, peak expiratory flow rate; l/s, liter per seconds.

### Predictors of pulmonary function test among PLHIV respondents

#### Simple linear regression presents predictors of FEV1_s_ (l) among PLHIV respondents

A simple linear regression was employed to determine the factors that predict the FEV1_s_ (l) of the PLHIV respondents. As a result, a simple linear regression model revealed a significant association (*p* < 0.25) between FEV1_s_ (l) and sex (male sex was used as a reference), height, respiratory symptoms (SGRQ score <24 being used as a reference), history of pulmonary TB (no. being used as a reference), duration of HIV infection, duration of treatment, treatment regimen (another treatment regimen being used as a reference), current CD4 cell count, and khat chewing (no. being used as a reference).

Conversely, age, weight, current viral load, BMI, drug adherence, alcohol taking, and smoking status were not statistically significant predictors of FEV1_s_ (l) (*p* > 0.25) among the PLHIV patients (Table 5).

**Table 5:** Predictors of FEV1_s_ among *PLHIV* respondents in jimma town, southwest Ethiopia, 2020.

PFT indices	Variables	*B*	95% CI	*P*-value	Adjusted *R*^2^
FEV1_s_ (l)
	Age	0.003	−0.014, 0.02	0.706	−0.009
	Sex(m)	0.495	0.261, 0.728	0.001*	0.15
	Height(m)	0.9	0.9, 2.01	0.001*	0.096
	Weight(kg)	0.006	−0.005, 0.016	0.281	0.002
	BMI(kg/m^2^)	−0.009	−0.043, 0.025	0.596	−0.008
	History of PTB	−0.273	−0.622, 0.076	0.123*	0.015
	Respiratory symptom	−0.468	−0.722, −0.213	0.001*	0.115
	Duration of treatment	−0.035	−0.06, −0.01	0.007*	0.066
	Duration of HIV	−0.033	−0.058, −0.007	0.012*	0.056
	Treatment regimen	0.539	0.191, 0.887	0.003*	0.082
	Smoking status	−0.079	−0.354, 1.95	0.568	0.007
	Alcohol taking	−0.045	−0.424, 0.334	0.814	−0.01
	Khat chewing	−0.281	−0.696, 0.125	0.17*	0.009
	Current CD4 count	0.0001	0.0001, 0.0002	0.215*	0.006
	Current viral load	0.0001	0.0001, 0.0003	0.272	0.002
	Drug adherence	−0.138	−0.393,0.116	0.283	0.002

Simple linear regression, PTB-pulmonary tuberculosis.

* *p* < 0.25 taken as significant predictors, TX, treatment; CD4, cluster of differentiation 4; FEV1, forced expiratory volume in the 1st second.

#### Multiple linear regression model presenting predictors of FEV1_s_ (l) among PLHIV respondents

Multiple linear regression analysis was utilized to obtain the best fit linear combination of sex, height, respiratory symptoms, duration of HIV infection, duration of treatment with HAART, treatment regimen, current CD4 count, history of pulmonary tuberculosis, and khat chewing, which were significant in simple linear regression (*p* < 0.25).

As a result, sex, respiratory symptoms, duration of HIV infection, duration of treatment with HAART, treatment regimen, and khat chewing were significant predictors of FEV1_s_ (l) (*p* < 0.05). Conversely, height, current CD4 count, and pulmonary tuberculosis were not significant predictors of FEV1_s_ (l) (*p* > 0.05). Being a female meant having FEV1_s_ (l) lower than 0.579 liters compared to being a male (*β *= 0.579, p0.001).

Respondents who reported respiratory symptoms decreased FEV1_s_ (l) by 0.461 liters compared to those devoid of respiratory symptoms (*β *= −0.461, p0.001). Similarly, respondents who reported chewing khat decreased their FEV1_s_ (l) by 0.453 liters compared to non-chewer respondents (*β *= −0.453, p0.012).

A unit increment in the duration of HIV infection resulted in a 0.08-liter decline in FEV1_s_ (*β *= −0.08, p0.03) when the history of pulmonary tuberculosis held constant. A unit increment in the duration of treatment resulted in a 0.09-liter decline in FEV1_s_ (l) (*β* = −0.09, p0.045). Similarly, respondents who were in the first-line treatment regimen had 0.324-liters higher FEV1_s_ (l) than respondents in other treatment regimens (*β*=0.324, p0.035) (Table 6).

**Table 6:** Predictors of FEV1 among *PLHIV* respondents in jimma town, southwest Ethiopia, 2020.

PFT indices	Variable	*Β*	95% CI	*P*-value	Adjusted *R*^2^
FEV1_s_ (l)					0.381
	Sex(m)	0.579	0.322,0.83	0.001*	
	Duration of HIV	−0.08	−0.006,0.173	0.03*	
	Duration of treatment	−0.09	−0.0173,−0.002	0.045*	
	Respiratory symptom	−0.461	−0.705,−0.218	0.001*	
	Current CD4 count	−0.005	−0.0001,0.005	0.854	
	Height(m)	0.458	−0.861,1.778	0.492	
	History of PTB	−0.066	−0.362,0.229	0.657	
	Khat chewing	−0.453	−0.802,−0.104	0.012*	
	Treatment regimen	0.324	0.024,0.624	0.035*	

Multiple linear regression.

* *p* < 0.05 taken as significant predictors, PTB, pulmonary tuberculosis; PFT, pulmonary function test; TX, treatment; RVI, retroviral infection; FEV1, forced expiratory volume in the 1st second; CD4, cluster of differentiation 4.

## Discussion

The mean percentage of the predicted FEV1 was significantly more reduced in the PLHIV participants than in the HIV-negative participants in the current study. This report is concordant with a study conducted in Copenhagen (17). This observation might be due to HIV persistently infecting alveolar macrophage—more often smaller alveolar macrophages (6). Conversely, this finding is contradicted by the research conducted in Amsterdam, Netherlands, which reported that the mean of FEV1 was not significantly different between PLHIV and HIV-negative respondents (18). The reason might be due to the relatively high smoking status and alcohol abuse in the controls (18). Moreover, other socio-demographic variations and methodological choices between the two groups might be perpetuating the difference.

Our findings revealed that the mean percentage of predicted FVC was significantly reduced in PLHIV participants than in HIV-negative respondents. This observation aligns with studies conducted in Cape Town, South Africa, and Amsterdam (18, 19). The concurrent decline in FEV1 and FVC with the slight preservation of the FEV1/FVC ratio was observed in our study. This might indicate the progressive development of isolated interstitial restrictive pulmonary diseases combined with obstructive pulmonary disease in PLHIV compared to HIV-negative patients. Apart from this, HIV status has been independently correlated with a higher probability of a fast decline in FEV1 and FVC with a slightly higher ratio of FEV1/FVC (20).

Being male was a positive predictor of FEV1 (l) in the current study. This finding was consistent with a study carried out in the United States (21). Apart from the physiological difference between male and female people, female people have more intensified CD8+ T cells and interferon-mediated immune activation at a given level of viremia than male people do (22). Our observation went against studies conducted in South Africa and Pittsburg (12, 23). Such a discrepancy might be due to variability in sample size, substance abuse, and occupational exposure between male and female people.

The current study also reported that the duration of HAART treatment was a negative predictor of FEV1 (l). This result is inconsistent with studies conducted in Minneapolis and Pittsburg, United States (21, 24). This might be due to HAART inducing oxidative stress by triggering the massive production of reactive oxygen species and inhibiting antioxidant production in the many cells of the pulmonary system (25). On the contrary, this finding went against a study conducted in Zimbabwe (26), which demonstrated that the early initiation of ART prevents the decline of FEV1. The reason is probably due to the difference in the duration of treatment and treatment regimen.

The present study also revealed that the duration of HIV infection is a negative predictor of FEV1. This observation is consistent with studies carried out in Zimbabwe and Denmark (16, 27). However, this finding went against research carried out in South Africa, which demonstrated that the duration of HIV has no association with the decline of pulmonary function in HIV-positive people (12). This is possibly due to variations in sample size, duration of HIV infection, and other HIV characteristics.

Similarly, the present study showed that the presence of respiratory symptoms is a negative predictor of FEV1 (l). This finding agrees with studies carried out in Nigeria and Baltimore (28, 29). It is probably because HIV as a virus is associated with persistent and consistent immune activation; HIV-activated deregulated adaptive immune response and lifetime low-grade CD8-T cell lymphocytic alveolitis might contribute to this association (29, 30).

Our findings also showed that khat chewing is a negative predictor of FEV1 (l). The association between khat chewing and pulmonary function impairment has not been elucidated very well. It is possible that the negative association between khat chewing and reduced pulmonary function test is probably because khat chewing could interfere with the drug adherence and feeding appetite (anorexia) of PLHIV.

Finally, the known influential predictors of FEV1 (age, height, and weight) had no significant association in the present study. It can be suggested that the PLHIV study respondents in our study had no big differences in terms of age, height, and weight that could possibly cause variation in FEV1 within the study group.

As part of our strength, this article is the first to study the effect of HIV infection on pulmonary function and to compare it with age- and sex-matched non-HIV-positive respondents. We extensively explore the potential risk factors that affect pulmonary function, including khat and alcohol use, HIV characteristics, drug adherence, and respiratory symptoms. The current study contributes its part to the present understanding of PLHIV and its impact on pulmonary function. Similarly, the current study highlights possible predictors of pulmonary function in PLHIV patients.

## Conclusion

Pulmonary functions were significantly reduced in PLHIV respondents compared to age- and sex-matched HIV-negative respondents. Sex, duration of HIV, duration of HAART treatment, khat chewing, respiratory symptoms, and treatment regimen were significant predictors of FEV1 (l) in PLHIV patients. Height, current viral load, current CD4 count, history of PTB, alcohol consumption, and degree of adherence had no significant association with FEV1 (l) in PLHIV participants. Based on the current results of this study, health providers should screen PLHIV patients with respiratory symptoms, prolonged duration of HIV infection, prolonged duration of treatment, and khat chewing for non-infectious lung disorders while treating them.

## Data Availability

The original contributions presented in the study are included in the article/Supplementary Material, further inquiries can be directed to the corresponding author/s.
